# The SNP rs931794 in 15q25.1 Is Associated with Lung Cancer Risk: A Hospital-Based Case-Control Study and Meta-Analysis

**DOI:** 10.1371/journal.pone.0128201

**Published:** 2015-06-16

**Authors:** Qi Wang, Juntao Ke, Qibin Song, Weiguo Hu, Xuzai Lu, Zhenling Wang, Hongyun Gong, Tangpeng Xu, Xueqin Chen, Bin Xu, Cheng Liu, Yun Sun, Yajie Gong, Yang Yang, Ying Zhu

**Affiliations:** 1 Department of Oncology, Renmin Hospital of Wuhan University, Wuhan, China; 2 Department of Epidemiology and Biostatistics, and the Ministry of Education Key Lab of Environment and Health, School of Public Health, Tongji Medical College, Huazhong University of Science and Technology, Wuhan, China; 3 Guangdong Maternal and Child Health Care Hospital, Guangzhou, China; 4 Department of hospital infection control, Tongji Hospital, Tongji Medical College, Huazhong University of Science and Technology, Wuhan, China; University of Hawaii Cancer Center, UNITED STATES

## Abstract

**Background:**

Lung cancer is one of the most common human malignant diseases and the leading cause of cancer death worldwide. The rs931794, a SNP located in 15q25.1, has been suggested to be associated with lung cancer risk. Nevertheless, several genetic association studies yielded controversial results.

**Methods and Findings:**

A hospital-based case-control study involving 611 cases and 1062 controls revealed the variant of rs931794 was related to increased lung cancer risk. Stratified analyses revealed the G allele was significantly associated with lung cancer risk among smokers. Following meta-analysis including 6616 cases and 7697 controls confirmed the relevance of rs931794 variant with increased lung cancer risk once again. Heterogeneity should be taken into account when interpreting the consequences. Stratified analysis found ethnicity, histological type and genotyping method were not the sources of between-study heterogeneity. Further sensitivity analysis revealed that the study “Hsiung et al (2010)” might be the major contributor to heterogeneity. Cumulative meta-analysis showed the trend was increasingly obvious with adding studies, confirming the significant association.

**Conclusions:**

Results from our current case-control study and meta-analysis offered insight of association between rs931794 and lung cancer risk, suggesting the variant of rs931794 might be related with increased lung cancer risk.

## Introduction

Lung cancer is one of the most common human malignant diseases and the leading cause of cancer-related death in western society. It accounts for 87697 deaths in males and 70389 deaths in females of American in 2009[1]. The incidence and mortality rates of lung cancer have increased rapidly in developing countries for the past few years. In China, the mortality rate of lung cancer is from 0.07‰ in 1970s to 0.4‰ in 2000[2]. Environment factors such as smoking, pollution and life style have been established to alter risk of cancer[3,4,5,6,7] Accumulative evidence indicated that tobacco smoking accounts for approximately 80% of lung cancer patients[8], but only a small fraction of heavy smokers develop lung cancer, suggesting the individual genetic factors may influence susceptibility to lung cancer.

A study investigated a high-risk lung cancer family and suggested the genes of familial lung cancer were located in 6q23-25[9]. Nonetheless, the result could not be the same in other high-risk families and approximately 1% of patients have explicit lung cancer family history. In recent years, a lot of studies were designed to screen the candidate susceptibility genes of lung cancer, and most of them focused on genes theoretically involved in cell growth, apoptosis and migration. Despite many attempts for the past years, the specific biomarkers for lung cancer risk were still not identified. Since studies of candidate genes have not got desired results, the researchers explored the contribution of common low-penetrance genes instead of high-penetrance genes. Genome-wide association study (GWAS) has made contributions to identification of genetic variants related to disease without comprehension of gene function. To date, several large GWAS of lung cancer have identified multiple common single nucleotide polymorphisms (SNPs) on chromosomes 15q25.1[10,11,12]. The SNPs of nicotenic acetylcholine receptor subunits in 15q25.1 have been confirmed to be in association with lung cancer risk. The rs931794, located in the aminoglycoside phosphotransferase domain containing 1 (*AGPHD1*) gene which is involved in the cluster of cholinergic nicotenic receptor subunit genes, has been supposed to be associated with lung cancer risk. A number of epidemiological studies have been conducted to examine the association between rs931794 and lung cancer risk in diverse populations[13,14,15,16]. However, the results of these studies are inconsistent. The difficulty of replication was partially due to the modest effect of rs931794 polymorphism. Besides, genetic association studies with limited population may have a lack of power to demonstrate the association. Nevertheless, meta-analysis, a statistical tool to combine data for more robust estimation, could clarify inconclusive results in genetic association studies. In this study, we sought to assess potential susceptibility of rs931794 with lung cancer risk. We designed a case-control study to evaluate the relationship in a Han Chinese population. Moreover, we conducted a meta-analysis, combining results from published articles and our current case-control study, to provide a more precise estimation of the association between rs931794 and lung cancer risk.

## Materials and Methods

### Study populations

The study adheres to the STREGA guidelines[17] for the reporting of genetic association studies. We enrolled a total of 611 new diagnosed lung cancer patients as cases from Tongji Hospital of Huazhong University of Science and Technology, Wuhan, China between 2009 and 2011. The written informed consent was obtained from each participant at the time of enrollment. Meanwhile, personal data about demographic features including age and gender, and potential influencing factors, such as smoking status, were perceived through questionnaires. All the cases had to have histologically confirmed lung cancer and not received any treatment before blood sample collection. The histologic types of lung cancer are categorized according to the World Health Organization classification system and classified as non-small cell lung cancer (NSCLC) and small cell lung cancer (SCLC)[18]. Controls were frequency matched with cases by sex and age (interval of 5 years). They were cancer-free individuals selected from medical examination program at the same hospital during the same period as the patients were enrolled, without history of serious respiratory disease and other cancer. Part of our controls overlapped with the subjects reported by Zhu et al[19]. The response rate for control subjects was 92.0%. The ethics committee of Tongji Hospital of Huazhong University of Science and Technology approved this study that was conducted in accordance with the guidelines outlined in Declaration of Helsinki. All participants were unrelated Han ethnic Chinese in the same region. A 5-ml peripheral blood sample was collected from all enrolled participants.

### DNA isolation and genotyping

Genomic DNA was isolated from peripheral blood sample using the RelaxGene Blood System DP319-02 (Tiangen, Beijing, China) according to the manufacturer’s instructions. The TaqMan SNP Genotyping Assay (Applied Biosystems, Foster city, CA) on a 7900HT Fast Real-Time PCR System (Applied Biosystems, Foster city, CA) were used to detect the polymorphism of rs931794. For quality control, 5% of duplicated samples were randomly sequenced in order to assess the reproducibility, with a concordance rate of 100%.

### Statistical analysis

The *t* test, Fisher exact test, and *χ* ^*2*^-test were applied to estimate differences in the distribution of demographic variables, potential influencing factors and genotypes between cases and controls, where appropriate. The association between rs931794 and lung cancer susceptibility was estimated by calculating odds ratio (OR) and 95% confidence interval (CI). The genotypic OR and its 95% CI were calculated after adjusting for age, sex and smoking status under logistic regression model. We defined the early-onset cases as who were < = 50.0 years of age in order to explore the association between the SNP and early-onset of cancer. To avoid the assumptions of genetic models, dominant, recessive and additive model for rs931794 in association with lung cancer were analyzed. Hardy-Weinberg equilibrium was assessed by the goodness-of-fit *χ* ^*2*^ test for genotypes in the control group[20]. All above statistical analysis were performed on the SPSS V12.0.

### Meta-analysis of rs931794 in association with lung cancer susceptibility

To further confirm the relevance of rs931974 with lung cancer susceptibility, a meta-analysis including published articles and our current studies was conducted. To ensure the rigour of this current meta-analysis, we designed and reported it according to the Preferred Reporting Items for Systematic Reviews and Meta-analyses (PRISMA) statement [21] and the checklist is shown in S1 Table. Systematic computerized searches of the PubMed, EMBASE and ISI Web of Science databases without language restriction were performed (up to March 7, 2015) by Q.Song and J. Ke. Following search terms were utilized ‘rs931794, AGPHD1, CHRNA5-CHRNA3-CHRNB4 or 15q25.1’ combined with ‘lung carcinoma, lung cancer’. The search was limited to human studies. All eligible studies were retrieved, and their bibliographies were checked for further relevant publications. Where insufficient data were available in trial publications and for unpublished trials, we contacted investigators in order to obtain data. The inclusion criteria were: (1) case-control or nested case-control study focused on the relationship between rs931794 and lung cancer risk; (2) Providing adequate data for calculating genotypic OR and corresponding 95% CI, including total number of lung cancer cases and controls, as same as the number of cases and controls for each genotypes; (3) Studies with full text articles. Exclusion criteria included: (1) reviews, tutorials and letters; (2) not case-control studies; (3) animal studies; (4) insufficient data were reported as number of cases and controls without genotype data; (5) duplicate data. When the same patient population was used in several publications, only the most recent, largest or complete study was selected. If more than one ethnic population were enrolled in one study, each population was considered as an independent study.

Information was carefully extracted from all eligible studies. Data extraction was done independently by two of the authors (Q. Wang and W. Hu). The following data were collected from each study: first author’s name, year of publication, country, ethnicity of the population, genotyping methods, histological type, gender, smoking status, OR observed in the primary studies, HWE (Hardy-Weinberg equilibrium), frequencies of genotypes in cases and controls. Two reviewers (J. Ke and X. Lu) independently performed the quality assessment with the Newcastle-Ottawa Scale[22] (NOS) for observation studies. NOS included 8 items which are categorized into three perspectives: selection of the study group, the comparability and exposure of interest. The quality of the studies was scored as follows: a maximum of 1 point for each item in the selection and exposure perspectives and a maximum of 2 points in the comparability. Disagreement was dealt with by discussion between the two authors. If the two authors could not reach a consensus, another author (Q. Song) was consulted to resolve the dispute and a final decision would follow by the majority of the votes.

Hardy-Weinberg equilibrium was firstly estimated in the controls for each study using *χ* ^*2*^ test at first, and p-value < 0.05 was considered as a significant disequilibrium [20]. ORs and corresponding 95%CIs were employed to assess the strength of associations between rs931794 and risk of lung cancer. The wild type AA was considered as a reference. The genetic comparisons included homozygous model (GG versus AA), heterozygous model (AG versus AA), dominant model (GG+AG versus AA), recessive model (GG versus AG+AA, with reference of AG+AA) and an additive “per-allele” model was also considered. For additive model, scores of 0, 1 and 2 were assigned to genotype GG, AG and AA, respectively. The per-allale ORs were calculated by logistic regression model. In this meta-analysis, we assessed heterogeneity by Cochran’s Q-test[23,24] and *I* ^*2*^ statistic[23]. Heterogeneity was considered significantly at *P*<0.1 of Cochran’s Q-test. The following thresholds were used to quantify *I* ^*2*^ metric: *I* ^*2*^=0-25%, no heterogeneity; *I* ^*2*^ = 25-50%, moderate heterogeneity; *I* ^*2*^ = 50–75%, large heterogeneity; *I* ^*2*^ =75–100%, extreme heterogeneity. If significant heterogeneity was detected (*P*<0.1), the random-effects model (the DerSimonian and Laird method)[25] instead of the fixed-effects model (the Mantel-Haenszel method)[26] was applied to combine data from studies. The square root of *tau* ^*2*^ was the standard deviation of underlying effects across studies and used to estimate the heterogeneity independent of sample size. The smaller the *tau* is, the less heterogeneity between studies. In order to explore the sources of between-study heterogeneity, we conducted stratified analysis according to ethnicity, histological type and genotyping method. For the purpose of assessing the impact of a single study on the pooled OR and confirming stability of the results, sensitivity analysis was performed to repeat analyses by sequential removal of individual studies[27]. Cumulative analysis was conducted to investigate the trend between rs931794 polymorphism and lung cancer risk with accumulation of studies by published year. Funnel plots and Egger’s test[28,29] were used to explore the presence of publication bias. The trim-and-fill method was implemented to evaluate number of potentially missing studies and assess the effect of publication bias on meta-analysis[30]. All *P* values are two-tailed with a significant level at 0.05, except Cochrane’s Q-test for heterogeneity (*P*<0.1). All the statistical tests used in our meta-analysis were performed with STATA version 10.0 (Stata Corporation, College Station, TX) and R 3.1.1 (R Development Core Team; Vienna, Austria).

## Results

### Results of case-control study

A total of 611 cases of lung cancer and age- and gender-matched 1062 controls were enrolled from 2009 to 2011. The relevant characteristics of subjects were listed in Table 1. As shown in Table 1, males were 68.6% in cases compared 70.2% in controls. Mean age was 61.5 years for cases and 61.0 years for controls. There was no significant difference between the case and control group in terms of gender (*P* = 0.475) and age (*P* = 0.155) distribution. More smokers were presented among cases compared with controls as expected (53.0% among cases versus 43.1% among controls, *P*<0.001). Among the cases, 427 (69.9%) were classified as NSCLC and 184 (30.1%) as small cell lung cancer.

**Table 1:** The characteristics of the study population.

	Case (N = 611)	Control (n = 1062)	
Variables	NO. (%)	NO. (%)	*P*
Gender			0.475^a^
Male	419(68.6)	746(70.2)	
Female	192(31.4)	316(29.8)	
Median age	61.5	61.0	0.311^b^
≤61.0	322(52.7)	531(50.0)	0.287^a^
>61.0	289(47.3)	531(50.0)	
Smoking status			<0.001^a^
Never-smoker	279(45.7)	599(56.4)	
Smoker	324(53.0)	458(43.1)	
Unknown	8(1.3)	5(0.5)	
Histological type			
NSCLC	427(69.9)		
Others	184(30.1)		

Abbreviations: NSCLC, non-small cell lung cancer.

^a^ *P* value was calculated by the *x* ^2^ test.

^b^ *P* value was calculated by the Wilcoxon test.

The frequencies of genotypes in the controls conformed to Hardy-Weinberg equilibrium (*P* = 0.921). However, the genotype distribution was markedly different between cases and controls (*P* = 0.001). Table 2 showed the distribution of rs931794 polymorphism in cases and controls. Logistic regression analysis indicated that the GG genotype carriers had 1.813-fold elevated risk of developing lung cancer compared with AA genotype carriers. We combined the GG with the AG into G carrier group in order to increase statistical power for evaluating relevance of rs931794 with lung cancer risk. The increased risk of lung cancer was discovered in dominant comparison, with adjusted OR was 1.250 (95%CI, 1.014–1.540). Moreover, the G allele was statistically significant with increased risk of lung cancer in the additive model and allelic model. We further represented analysis confined to cases with histologically confirmed NSCLC. Similar significant relation and weaker genetic effect were found in NSCLC. Although dominant model failed to demonstrate the association between rs931794 polymorphism and lung cancer risk, significantly increased risk of lung cancer was found in the additive model (adjusted OR, 1.269; 95%CI, 1.071–1.505) and allelic model (adjusted OR, 1.258; 95%CI, 1.056–1.497).

**Table 2:** Association between rs931794 and lung cancer risk in a Chinese population.

Genotype	Controls (n = 1062)N(%)	Cases (n = 611)N(%)	*P* ^*a*^	Crude OR (95%CI)	*p*	Adjusted OR (95%CI)^b^	*P* ^b^
Successful genotype	1058(99.6)	562(92.0)					
*Overall*							
AA	503(47.5)	235(41.8)		1.000		1.000	
AG	452(42.7)	239(42.5)		1.132(0.908, 1.411)	0.271	1.121(0.897, 1.401)	0.315
GG	103(9.7)	88(15.7)	0.001	**1.829(1.323, 2.528)**	**<0.001**	**1.813(1.306, 2.517)**	**<0.001**
Dominant model				**1.261(1.026, 1.550)**	**0.028**	**1.250(1.014, 1.540)**	**0.037**
Recessive model				**1.721(1.269, 2.335)**	**<0.001**	**1.174(1.259, 2.335)**	**0.001**
Allele A	1458(68.9)	709(63.1)		1.000		1.000	
Allele G	658(31.1)	415(36.9)	0.001	**1.297(1.114, 1.510)**	**0.001**	**1.287(1.103, 1.501)**	**0.001**
Additive model				**1.286(1.107, 1.494)**	**0.001**		
*NSCLC*							
AA	503(47.5)	165(42.2)		1.000		1.000	
AG	452(42.7)	166(42.5)		1.120(0.872, 1.438)	0.376	1.102(0.856, 1.420)	0.450
GG	103(9.7)	60(15.3)	0.008	**1.776(1.234, 2.555)**	**0.002**	**1.736(1.201, 2.508)**	**0.003**
Dominant model				1.241(0.982, 1.569)	0.070	1.221(0.963, 1.547)	0.099
Recessive model				**1.681(1.194, 2.366)**	**0.003**	**1.655(1.170, 2.340)**	**0.004**
Allele A	1458(68.9)	496(63.4)		1.000		1.000	
Allele G	658(31.1)	286(36.6)	0.005	**1.278(1.076, 1.518)**	**0.005**	**1.258(1.056, 1.497)**	**0.010**
Additive model				**1.269(1.071, 1.505)**	**0.006**		

Abbreviations: OR, Odds ratio; 95%CI, 95% confidence interval.

^a^ *P* values were calculated by the Pearson Chi-Square test.

^b^ Data were calculated by logistic regression model after adjusting for age, sex, and smoking status.

The relevance of rs931794 genotypes with lung cancer risk was further examined with stratifying by smoking status, age and gender (see S2 Table). Among non-smokers, OR of per-G allele was 1.237 (95%CI, 0.998–1.534) under additive model. However, among smokers, increased risk of lung cancer was observed among G allele carriers with per-G OR of 1.314 (95%CI, 1.061–1.627). When stratified by the median age (age <= 61.0, age>61.0), significant association of rs931794 with lung cancer risk was found in both age groups under the additive model. The rs931794 was related with risk of lung cancer among people more than 50 years old. The relation was not noteworthy among people younger than 50 years old (OR, 1.378; 95%CI, 0.919–2.067). According to gender, no association between rs931794 and lung cancer susceptibility was observed in females, but the relevance of the rs931794 with lung cancer susceptibility was significant in males (OR, 1.294; 95%CI, 1.078–1.553).

### Results of meta-analysis

#### Study characteristics

After exclusion of duplicate and irrelevant studies (see S1 Fig), 4 published articles[13,14,15,16] plus current study, including 6616 cases and 7697 controls exploring the relation between rs931794 and lung cancer susceptibility were identified according to the inclusion criteria in the meta-analysis. The references of the flow chart were placed in S1 Appendix. Another two large GWAS, Hung[10] and Thorgeirsson[11] were excluded due to their insufficient data for calculation of ORs after we failed to obtain necessary data from corresponding authors by contacting them through e-mails. Schwartz[13] assessed relation of rs931794 polymorphism with lung cancer risk and provided data into Caucasians and African-American population, so we divided it into two studies. Of the 6 studies, 4 studies were performed among Asians, 1 study among Caucasians and 1 study among African-American population. Most of the included studies detected the rs931794 polymorphism using Taqman assay except Truong[15]. Two of the included studies only enrolled patients with NSCLC, while others enrolled patients without histological restriction. Genotypes of rs931794 in controls were accorded with Hardy-Weinberg equilibrium for all included studies. Detailed information of these articles was listed in Table 3. The estimated score of included studies ranged from 6 to 7 (the detailed information showed in S3 Table).

**Table 3:** The characteristics of included studies.

First author	Year	Country	Ethnicity	Genotyping method	Histological type	Case/Control(Never smoked)	Case/Control(Male)	Case/Control(Total)	OR(95%CI)	HWE *P*
		
	
Schwartz-W	2009	USA	Caucasian	Taqman	NSCLC	111/451	—	630/835	1.25(1.06-1.46) ^a^	0.09
Schwartz-AA	2009	USA	African-American	Taqman	NSCLC	41/196	—	402/435	1.41(1.10-1.81) ^a^	0.40
Truong	2010	France	Asian	Taqman	Lung Cancer	674/1270	838/902	1694/2126	1.03(0.93-1.14) ^a^	0.10
				&Illumina						
Hsiung	2010	China	Asian	Taqman	Lung Cancer	2563/2541	0/0	2563/2541	1.15(0.97-1.36) ^b^	0.22
Ito	2012	Japan	Asian	Taqman	Lung Cancer	177/290	531/531	716/716	1.15(0.98-1.35) ^a^	0.29
Current Study	2014	China	Asian	Taqman	Lung Cancer	279/599	419/746	611/1062	1.29(1.11, 1.49) ^a^	0.92

Abbreviations: NSCLC, non-small cell lung cancer; OR, Odds ratio; 95%CI, 95% confidence interval; HWE, Hardy-Weinberg equilibrium.

^a^ OR was calculated under additive model.

^b^ OR was calculated under dominant model.

#### Overall meta-analysis of rs931794 in relevance of lung cancer risk

**Fig 1:**
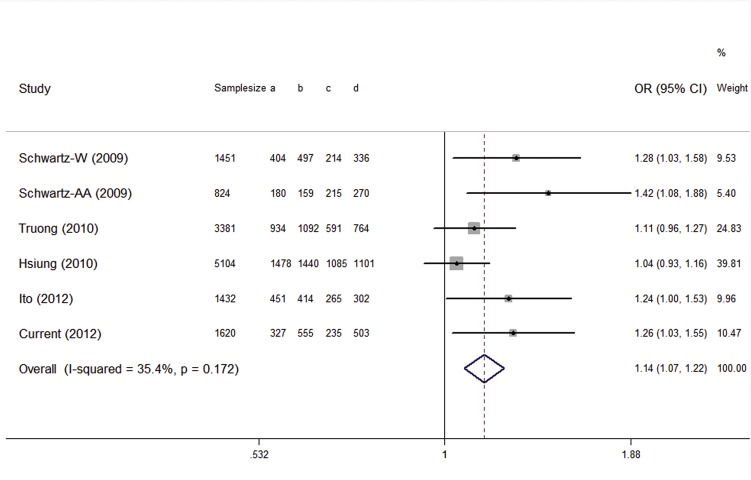
The forest plots of OR with 95%CI for the rs931794 with lung cancer risk under dominant model. Fixed-effects pooled OR=1.14, 95%CI = 1.07-1.22, P<0.001; P_heterogeneity_ = 0.172.

Table 4 listed the main consequences of the pooled analyses. There was significant heterogeneity in all genetic comparisons except heterozygous and dominant comparison. The random-effects model was utilized to pool data when significant heterogeneity existed. Overall, relevance of rs931794 variant with increased risk of lung cancer was observed in dominant model (OR, 1.143; 95%CI, 1.068-1.224; shown in Fig 1). Moreover, the G allele was statistically significant with increased risk of lung cancer in the homozygous, heterozygous, additive model and allelic model. The meta-analysis revealed similar results to our case-control study.

**Table 4:** Meta-analysis of the rs931794 in association with lung cancer risk.

Category	Genetic model	OR(95%CI)	*P*	*I* ^*2*^	*tau*	*P* for heterogeneity
Overall	AG/AA	1.141(1.061-1.227)	<0.001	0.0	<0.0001	0.617
(n = 6)	GG/AA	1.263(1.007-1.583)	0.043	73.6	0.234	0.002
	Dominant model	1.143(1.068-1.224)	<0.001	35.4	0.066	0.172
	Recessive model	1.166(0.947-1.437)	0.148	72.8	0.214	0.002
	G/A	1.144(1.038-1.262)	0.007	69.7	0.099	0.006
	Additive model	1.139(1.036-1.252)	0.007	68.1	0.095	0.008
*Ethnicity* **/** *Histological type*						
Asian (n = 4) **/**	AG/AA	1.114(1.029-1.206)	0.007	0.0	<0.0001	0.853
Lung cancer	GG/AA	1.209(0.911-1.603)	0.189	80.9	0.256	0.001
(n = 4)	Dominant model	1.111(1.031-1.197)	0.006	21.2	0.041	0.283
	Recessive model	1.134(0.868-1.481)	0.356	81.4	0.243	0.001
	G/A	1.107(0.989-1.239)	0.077	73.5	0.097	0.010
	Additive model	1.103(0.989-1.230)	0.079	72.3	0.093	0.013
*Genotyping method*						
Taqman	AG/AA	1.146(1.054-1.246)	0.001	0.0	<0.0001	0.478
(n = 5)	GG/AA	1.336(0.997-1.790)	0.052	77.6	0.285	0.001
	Dominant model	1.156(1.068-1.250)	<0.001	46.2	0.090	0.114
	Recessive model	1.228(0.939-1.607)	0.134	76.8	0.260	0.002
	G/A	1.176(1.037-1.334)	0.012	74.3	0.121	0.004
	Additive model	1.169(1.035-1.320)	0.012	72.9	0.116	0.005

Abbreviations: OR, Odds ratio; 95%CI, 95% confidence interval.

#### Stratified analysis

There was significant heterogeneity in meta-analysis. Asians were enrolled in four studies, Caucasians and African-American race population each were enrolled in one study. After stratifying by ethnicity, significant heterogeneity still existed under homozygous, recessive, additive and allelic models. In stratified analysis based on histological type, the studies enrolled patients without histological restriction were all from Asians, so the results were the same as analysis stratified by ethnicity. Studies enrolled NSCLC cases were not pooled in stratified analysis due to limited number of studies. According to genotyping method, heterogeneity was still not removed, indicating genotyping method was unlikely the source of heterogeneity.

#### Sensitivity analysis and cumulative meta-analysis

Since significant heterogeneity existed in meta-analyses, sensitivity analysis was performed to repeat analyses by sequential removal of individual studies so as to examine the influence of a single study on the pooled estimation and assess stability of the result. The detailed information of sensitivity analysis under dominant model was listed in Table 5. The sensitivity analysis showed the pooled ORs and 95% CIs were not changed before and after excluding of each study in dominant comparison, indicating the robustness of the current meta-analysis (see in S2 Fig). Hsiung[16] was the major contributor of heterogeneity in dominant model (*I* ^*2*^ = 35.4%, *P* = 0.172), after removal of the study, the heterogeneity greatly reduced (*I* ^*2*^ = 0.0%, *P* = 0.499). It could be accounted for different inclusion criteria among the included studies. Since Hsiung enrolled lung cancer cases from never-smoking females with adenocarcinoma, while others recruited patients without such strict criteria. Similar result was seen in additive comparison that no single study changed the pooled OR markedly and Hsiung was the contributor to heterogeneity.

**Table 5:** Sensitivity analysis of dominant model.

Study omitted	OR (95%CI)	*P*	*P* for heterogeneity	*I* ^*2*^	*tau*
Schwartz-W 2009	1.129 (1.051-1.214)	0.001	0.157	39.6%	0.071
Schwartz-AA 2009	1.127 (1.051-1.210)	0.001	0.265	23.5%	0.045
Truong 2010	1.156 (1.068-1.250)	<0.001	0.114	46.2%	0.089
Hsiung 2010	1.211 (1.110-1.321)	<0.001	0.499	0.0%	0
Ito 2012	1.132 (1.053-1.217)	0.001	0.131	43.5%	0.077
Current Study 2012	1.129 (1.051-1.214)	0.001	0.149	40.8%	0.071

Abbreviations: OR, Odds ratio; 95%CI, 95% confidence interval.

**Fig 2:**
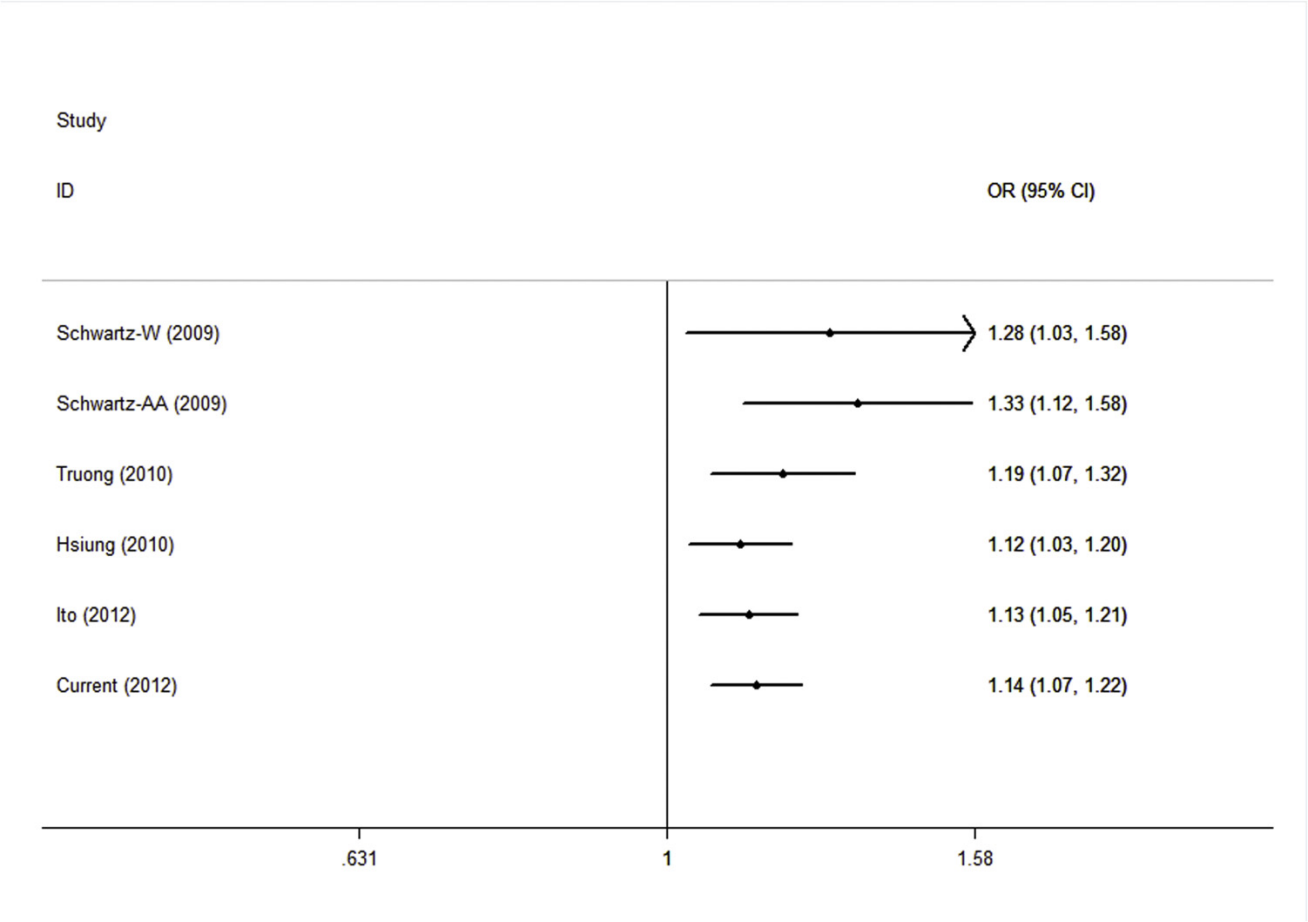
The cumulative meta-analysis of association rs931794 with lung cancer risk under dominant model.

The cumulative meta-analysis was performed to investigate the trend between rs931794 polymorphism and lung cancer risk with accumulation of studies by published year. As shown in Fig 2, the tendency toward significant relation became increasingly obvious over time in the dominant model. Meanwhile, the 95%CI for the pooled OR was narrower with the increase in the number of studies, suggesting the result was more and more precise with continually adding studies. Similar results were seen in other genetic comparisons.

#### Publication Bias

**Fig 3:**
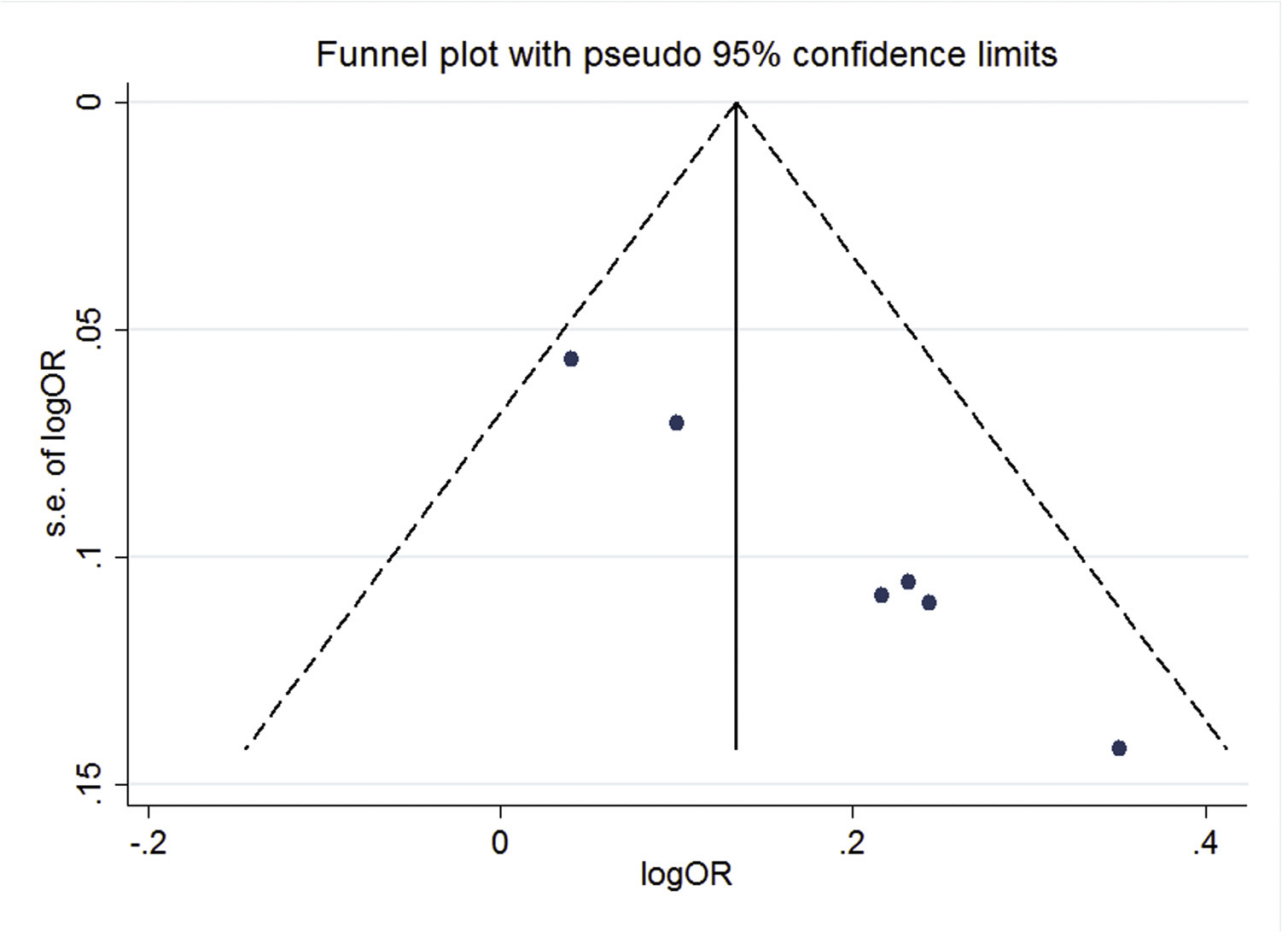
The overall funnel plot under dominant model.

The funnel plot of dominant model showed asymmetric distribution of the included studies, indicating there might be some unpublished articles were not included in the meta-analysis (shown in Fig 3). We included 5 studies because of strict inclusion criteria. Sterne et al [31] suggested only applying tests of funnel plot asymmetry methods if there are ten or more independent studies. The Cochrane Handbook for Systematic Reviews of Interventions[32] also suggested these methods should be used only when there were at least 10 studies included in the meta-analysis, because when there were fewer studies the power of the tests is too low to distinguish chance from real asymmetry. In order to maintain the integrity and provide readers with fully understanding of our meta-analysis, the results of Egger’s test and trim and fill method were placed in the supporting information (shown in S4 Table).

## Discussion

Due to the inconsistent results of the association between rs931794 and lung cancer risk reported by different researchers, we conducted a case-control study and meta-analysis to clarify the real relationship between the rs931794 genotypes and lung cancer risk. In this study, we found rs931794 was related with lung cancer risk in the case-control study performed in the Chinese population. Further meta-analysis combining data from published articles and our current studies showed this SNP might be related with lung cancer risk. To the best of our knowledge, this meta-analysis was the first study combining published articles to represent the association of rs931794 with lung cancer risk.

*CHRNA5-CHRNA3-CHRNB4*, the nicotenic acetylcholine receptor subunits, encode proteins that form receptors expressed in neurons and other tissues, particularly respiratory epithelium, pulmonary neuroendocrine cells and lung cancer cells[33]. The receptors bind to nicotine, N’-nitrosonornicotine and other potential carcinogens, leading to genetic mutations and neoplastic transformation[34]. SNPs in *CHRNA5-CHRNA3-CHRNB4* could be able to influence the binding function, thus influence individual lung cancer risk. Besides, Tournier[35] found nocitinic acetylcholine receptors actively contributed to the wound repair process of the respiratory epithelial cells by modulating intracellular calcium in cells. Krais and his colleagues[36] found nicotinic acetylcholine receptors also played an important role in carcinogenesis by modulating adhesion and motility in respiratory epithelial cells. It is suggested that SNPs in *CHRNA5-CHRNA3-CHRNB4* clusters of neotenic acetylcholine receptor subunit genes could influence individual cancer susceptibility by altering receptor nicotine binding function, normal cell proliferation, cell migration and wound repair. *AGPHD1* gene is involved in *CHRNA5-CHRNA3-CHRNB4* clusters and supposed to be associated with lung cancer risk. The rs931794, which belongs to *AGPHD1* gene, is located at 15q25.1 and has been identified as putative genes involved in susceptibility of lung cancer. The rs931794 is situated in the intronic region of *AGPHD1*, might play an important role in transcription regulation and thus impact on the *AGPHD1* expression. Although the *AGPHD1* in vivo function is unknown and no functional report is concerning the rs931794, we propose the hypothesis based on previous studies that the rs931794 might alter activity of certain members of neotenic acetylcholine receptors and thus affect lung cancer incidence. However, several genetic association studies reached separate conclusions for the past few years.

In this study, we firstly conducted a case-control study of the Chinese population. Logistic regression analysis revealed that the increased risk was in relation with the GG carriers compared with the AA carriers, and the similar results were also found under dominant, recessive, additive and allelic models. Further analysis confined with histologically confirmed NSCLC cases found weaker genetic effect and significant association of rs931794 with lung cancer risk. Stratified analyses according to smoking status, age and gender were conducted. The results showed the G allele was associated with lung cancer risk among smokers. It was strange that the variant allele tended to involve in lung cancer risk in non-smokers although the trend did not reach significant. This result could be accounted for the relative small sample size. However, there were several studies revealed the genetic involvement of rs931794 in lung cancer risk among smokers instead of non-smokers. Schwartz and colleagues[13] found the variant of rs931794 was not significantly associated with lung cancer risk in never smokers among Caucasians and African Americans. Nevertheless, risk was significantly related with rs931794 in ever smokers. Among Asian never-smoking females, Hsiung[16] found that risk was not statistically significant with the variant. Ito and colleagues[14] reached the same conclusion among Japanese. The meta-analysis conducted by M Gu[37] found *AGPHD1* rs8034191 was risk-conferring factors for lung cancer. The meta-regression analysis revealed an association of the polymorphism with lung cancer in patients with higher smoking rate. Several studies have revealed that the *CHRNA5-CHRNA3-CHRNB4* cluster of neotenic acetylcholine receptor subunit genes also have a role in nicotine dependence[38,39,40], and the variant in this genetic region might increase lung cancer risk through smoking[40,41]. The significant association among smokers might be due to the rs931794 modulates smoking behavior and thus increases lung cancer risk indirectly. Since males were more likely to be smokers, the relation of rs931794 with lung cancer risk was significant among males, but not among females. Regarding age range, rs931794 was related with risk of lung cancer among people more than 50 years old, but the relation was not significant among people younger than 50 years old. It is not accorded with the viewpoint that genetic susceptibility is related to early onset of disease. As mentioned above, SNPs in the *CHRNA5-CHRNA3-CHRNB4* cluster of neotenic acetylcholine receptor subunit genes could affect individual lung cancer susceptibility directly by influencing mucosa repair or indirectly by impacting on smoking behavior. Either way, these SNPs play an important role in exogenous carcinogens-induced carcinogenesis, which takes time to be carried out. This might be why the relation of rs931794 with lung cancer risk was positive among the older group, but not younger group.

The following meta-analysis including 6616 cases and 7697 controls combined previously published articles and our current study was conducted for the purpose of estimating the real relation between rs931794 and lung cancer risk. Overall results suggested all the genetic models could confer G allele in relevance of lung cancer risk, except recessive model. The meta-analysis of rs931794 proved the similar relation with lung cancer risk to the current case-control study. However, obvious between-study heterogeneity in the meta-analysis should be considered when explaining results and drawing conclusions. Heterogeneity was not reduced in further stratified analysis according to ethnicity, histological type and genotyping method under homozygous, recessive, additive and allelic model. After stratifying by ethnicity and histological type, heterogeneity was existed, but the variant of rs931794 was related to increased lung cancer as before. In stratified analysis based on genotyping method, heterogeneity was not removed. The stratified analysis suggested that ethnicity, histological type and genotyping method were not the major sources of between-study heterogeneity. Further sensitivity analysis found the main source of heterogeneity in the dominant model was the study conducted by Hsiung[16] and his colleagues. The heterogeneity was reduced after removal of this study, it could be explained that Hsiung enrolled lung cancer cases from never-smoking females suffered from adenocarcinoma and other studies enrolled cases without such rigorous set of inclusion criteria. Sensitivity analyses also revealed the pooled ORs were not changed before and after excluding of each study, indicating the results were persistent. Cumulative analysis was further performed to confirm the significant association, revealing the effect of G allele was more and more evident with the accumulation of more data by the published time.

Despite the strength of our study that yielded enough power to implement a comprehensive analysis, that’s a lot of room for improvement. At first, the sample size of the current case-control study was relatively small. So we conducted further meta-analysis. The following meta-analysis came to the same conclusion as our case-control study. Though sensitivity analysis and cumulative analysis showed stability of our results, the funnel plot indicated there might be some unpublished articles were not included in the meta-analysis. In addition, lung cancer is a complex disease, which is resulted from interactions between environmental carcinogens, such as nicotine, and genetic factors. Nevertheless, lacking individual personal data and environmental data limited us to further assess gene-gene and gene-environment interaction in the meta-analysis. Further studies should focus on the mechanism of lung cancer risk, especially gene-gene and gene-environment interactions.

In conclusion, the results from our current case-control study in the Chinese population and meta-analysis provided a comprehensive description of association between rs931794 and lung cancer risk, suggesting the variant of rs931794 might be related with increased lung cancer risk. However, more studies should be performed to confirm the authenticity of our conclusion. Since other factors, such as genetic background is also a predisposing factor of lung cancer, the interations between gene-gene and gene-environment should be carried on research for understanding the mechanism of lung cancer. Moreover, fine-mapping of 15q25.1 region or functional analysis should be conducted to identify more possible variants.

## Supporting Information

S1 Appendix The references of the flow chart. (DOCX)

S1 Fig Flow chart of study selection. (TIF)

S2 Fig The forest plots of sensitivity analysis under dominant model. (TIF)

S1 Table PRISMA Checklist. (DOCX)

S2 Table The association between rs931794 and risk of lung cancer by smoking status, median age, age range and sex. (DOCX)

S3 Table Quality assessment of included studies. (DOCX)

S4 Table Egger’s test and Nonparametric trim and fill analysis of publication bias in meta-analysis. (DOCX)
