# Supplementary material for: The SNP rs931794 in 15q25.1 Is Associated with Lung Cancer Risk: A Hospital-Based Case-Control Study and Meta-Analysis
Source: PLoS One. 2015 Jun 16;10(6):e0128201. doi: 10.1371/journal.pone.0128201 (PMC4469418; doi:10.1371/journal.pone.0128201)
Supplement: S1 Appendix — (DOCX) [file pone.0128201.s001.docx]

SI References:

1. Gu M, Dong X, Zhang X, Wang X, Qi Y, Yu J, et al. (2012) Strong association between two polymorphisms on 15q25.1 and lung cancer risk: a meta-analysis. PLoS One 7: e37970.

2. Nakamura H (2011) Genetics of COPD. Allergol Int 60: 253-258.

3. Yokota J, Shiraishi K, Kohno T (2010) Genetic basis for susceptibility to lung cancer: Recent progress and future directions. Adv Cancer Res 109: 51-72.

4. Brennan P, Hainaut P, Boffetta P (2011) Genetics of lung-cancer susceptibility. Lancet Oncol 12: 399-408.

5. Saccone NL, Culverhouse RC, Schwantes-An TH, Cannon DS, Chen X, Cichon S, et al. (2010) Multiple independent loci at chromosome 15q25.1 affect smoking quantity: a meta-analysis and comparison with lung cancer and COPD. PLoS Genet 6.

6. Galvan A, Dragani TA (2010) Nicotine dependence may link the 15q25 locus to lung cancer risk. Carcinogenesis 31: 331-333.

7. Niu XM, Lu S (2014) Acetylcholine receptor pathway in lung cancer: New twists to an old story. World J Clin Oncol 5: 667-676.

8. Scherf DB, Sarkisyan N, Jacobsson H, Claus R, Bermejo JL, Peil B, et al. (2013) Epigenetic screen identifies genotype-specific promoter DNA methylation and oncogenic potential of CHRNB4. Oncogene 32: 3329-3338.

9. Krais AM, Hautefeuille AH, Cros MP, Krutovskikh V, Tournier JM, Birembaut P, et al. (2011) CHRNA5 as negative regulator of nicotine signaling in normal and cancer bronchial cells: effects on motility, migration and p63 expression. Carcinogenesis 32: 1388-1395.

10. Falvella FS, Galvan A, Colombo F, Frullanti E, Pastorino U, Dragani TA. (2010) Promoter polymorphisms and transcript levels of nicotinic receptor CHRNA5. J Natl Cancer Inst 102: 1366-1370.

11. Paliwal A, Vaissiere T, Krais A, Cuenin C, Cros MP, Zaridze D, et al. (2010) Aberrant DNA methylation links cancer susceptibility locus 15q25.1 to apoptotic regulation and lung cancer. Cancer Res 70: 2779-2788.

12. Wu H, Wang Y, Wang S, Jiang M, Wang C, Fu W, et al. (2013) Is susceptibility locus for lung cancer in the 15q25 nicotinic acetylcholine receptor gene cluster CHRNA5-A3-B4 associated with risk of gastric cancer? Med Oncol 30: 576.

13. Gabrielsen ME, Romundstad P, Langhammer A, Krokan HE, Skorpen F (2013) Association between a 15q25 gene variant, nicotine-related habits, lung cancer and COPD among 56,307 individuals from the HUNT study in Norway. Eur J Hum Genet 21: 1293-1299.

14. Wang H, Zhao Y, Ma J, Zhang G, Mu Y, Qi G, et al. (2013) The genetic variant rs401681C/T is associated with the risk of non-small cell lung cancer in a Chinese mainland population. Genet Mol Res 12: 67-73.

15. Lococo F, Cesario A, Petracca-Ciavarella L, Granone P, Russo P (2012) Role of CHRNA5-A3 genetic Locus variants and developing drug for chronic obstructive pulmonary disease. Curr Med Chem 19: 5863-5870.

16. Kaakinen M, Ducci F, Sillanpaa MJ, Laara E, Jarvelin MR (2012) Associations between variation in CHRNA5-CHRNA3-CHRNB4, body mass index and blood pressure in the Northern Finland Birth Cohort 1966. PLoS One 7: e46557.

17. Tekpli X, Landvik NE, Skaug V, Gulsvik A, Haugen A, Zienolddiny S. (2013) Functional effect of polymorphisms in 15q25 locus on CHRNA5 mRNA, bulky DNA adducts and TP53 mutations. Int J Cancer 132: 1811-1820.

18. Jin G, Bae EY, Yang E, Lee EB, Lee WK, Choi JE, et al. (2012) A functional polymorphism on chromosome 15q25 associated with survival of early stage non-small-cell lung cancer. J Thorac Oncol 7: 808-814.

19. Munafo MR, Timofeeva MN, Morris RW, Prieto-Merino D, Sattar N, Brennan P, et al. (2012) Association between genetic variants on chromosome 15q25 locus and objective measures of tobacco exposure. J Natl Cancer Inst 104: 740-748.

20. Kapoor M, Wang JC, Bertelsen S, Bucholz K, Budde JP, Hinrichs A, et al. (2012) Variants located upstream of CHRNB4 on chromosome 15q25.1 are associated with age at onset of daily smoking and habitual smoking. PLoS One 7: e33513.

21. Yu K, Wacholder S, Wheeler W, Wang Z, Caporaso N, Landi, MT, et al. (2012) A flexible Bayesian model for studying gene-environment interaction. PLoS Genet 8: e1002482.

22. Wei C, Han Y, Spitz MR, Wu X, Chancoco H, Akiva P, et al. (2011) A case-control study of a sex-specific association between a 15q25 variant and lung cancer risk. Cancer Epidemiol Biomarkers Prev 20: 2603-2609.

23. Wojas-Krawczyk K, Krawczyk P, Biernacka B, Grzybek M, Kolodziej P, Kucharczyk T, et al. (2012) The polymorphism of the CHRNA5 gene and the strength of nicotine addiction in lung cancer and COPD patients. Eur J Cancer Prev 21: 111-117.

24. Xun WW, Brennan P, Tjonneland A, Vogel U, Overvad K, Kaaks R, et al. (2011) Single-nucleotide polymorphisms (5p15.33, 15q25.1, 6p22.1, 6q27 and 7p15.3) and lung cancer survival in the European Prospective Investigation into Cancer and Nutrition (EPIC). Mutagenesis 26: 657-666.

25. Chen J, Wu X, Pande M, Amos CI, Killary AM, Sen S, et al. (2011) Susceptibility locus for lung cancer at 15q25.1 is not associated with risk of pancreatic cancer. Pancreas 40: 872-875.

26. Kaur-Knudsen D, Bojesen SE, Tybjaerg-Hansen A, Nordestgaard BG (2011) Nicotinic acetylcholine receptor polymorphism, smoking behavior, and tobacco-related cancer and lung and cardiovascular diseases: a cohort study. J Clin Oncol 29: 2875-2882.

27. Wang Y, Broderick P, Matakidou A, Eisen T, Houlston RS (2011) Chromosome 15q25 (CHRNA3-CHRNA5) variation impacts indirectly on lung cancer risk. PLoS One 6: e19085.

28. Chen D, Truong T, Gaborieau V, Byrnes G, Chabrier A, Chuang SC, et al. (2011) A sex-specific association between a 15q25 variant and upper aerodigestive tract cancers. Cancer Epidemiol Biomarkers Prev 20: 658-664.

29. Gago-Dominguez M, Jiang X, Conti DV, Castelao JE, Stern MC, Cortessis VK, et al. (2011) Genetic variations on chromosomes 5p15 and 15q25 and bladder cancer risk: findings from the Los Angeles-Shanghai bladder case-control study. Carcinogenesis 32: 197-202.

30. Erlich PM, Hoffman SN, Rukstalis M, Han JJ, Chu X, Linda Kao WH, et al. (2010) Nicotinic acetylcholine receptor genes on chromosome 15q25.1 are associated with nicotine and opioid dependence severity. Hum Genet 128: 491-499.

31. Saccone NL, Schwantes-An TH, Wang JC, Grucza RA, Breslau N, Hatsukami D, et al. (2010) Multiple cholinergic nicotinic receptor genes affect nicotine dependence risk in African and European Americans. Genes Brain Behav 9: 741-750.

32. Lips EH, Gaborieau V, McKay JD, Chabrier A, Hung RJ, Hashibe M, et al. (2010) Association between a 15q25 gene variant, smoking quantity and tobacco-related cancers among 17 000 individuals. Int J Epidemiol 39: 563-577.

33. Wang JC, Bierut LJ, Goate AM (2009) Variants weakly correlated with CHRNA5 D398N polymorphism should be considered in transcriptional deregulation at the 15q25 locus associated with lung cancer risk. Clin Cancer Res 15: 5599, 5599.

34. Zienolddiny S, Skaug V, Landvik NE, Ryberg D, Phillips DH, Houlston R, et al. (2009) The TERT-CLPTM1L lung cancer susceptibility variant associates with higher DNA adduct formation in the lung. Carcinogenesis 30: 1368-1371.

35. Falvella FS, Galvan A, Frullanti E, Spinola M, Calabro E, Carbone A, et al. (2009) Transcription deregulation at the 15q25 locus in association with lung adenocarcinoma risk. Clin Cancer Res 15: 1837-1842.

36. Le Marchand L, Derby KS, Murphy SE, Hecht SS, Hatsukami D, Carmella SG, et al. (2008) Smokers with the CHRNA lung cancer-associated variants are exposed to higher levels of nicotine equivalents and a carcinogenic tobacco-specific nitrosamine. Cancer Res 68: 9137-9140.

37. Young RP, Hopkins RJ, Hay BA, Epton MJ, Black PN, Gamble GD. (2008) Lung cancer gene associated with COPD: triple whammy or possible confounding effect? Eur Respir J 32: 1158-1164.

38. VanderWeele TJ, Asomaning K, Tchetgen TE, Han Y, Spitz MR, Shete S, et al. (2012) Genetic variants on 15q25.1, smoking, and lung cancer: an assessment of mediation and interaction. Am J Epidemiol 175: 1013-1020.

39. Wang Y, Broderick P, Matakidou A, Eisen T, Houlston RS (2010) Role of 5p15.33 (TERT-CLPTM1L), 6p21.33 and 15q25.1 (CHRNA5-CHRNA3) variation and lung cancer risk in never-smokers. Carcinogenesis 31: 234-238.

40. Bloom AJ, Hartz SM, Baker TB, Chen LS, Piper ME, Fox L, et al. (2014) Beyond cigarettes per day. A genome-wide association study of the biomarker carbon monoxide. Ann Am Thorac Soc 11: 1003-1010.

41. Stephens SH, Hartz SM, Hoft NR, Saccone NL, Corley RC, Hewitt JK, et al. (2013) Distinct loci in the CHRNA5/CHRNA3/CHRNB4 gene cluster are associated with onset of regular smoking. Genet Epidemiol 37: 846-859.

42. Flora AV, Zambrano CA, Gallego X, Miyamoto JH, Johnson KA, Cowan KA, et al. (2013) Functional characterization of SNPs in CHRNA3/B4 intergenic region associated with drug behaviors. Brain Res 1529: 1-15.

43. Ren JH, Jin M, He WS, Liu CW, Jiang S, Chen WH, et al. (2013) Association between CHRNA3 rs1051730 genotype and lung cancer risk in Chinese Han population: a case-control study. J Huazhong Univ Sci Technolog Med Sci 33: 897-901.

44. He P, Yang XX, He XQ, Chen J, Li FX, Gu X, et al. (2014) CHRNA3 polymorphism modifies lung adenocarcinoma risk in the Chinese Han population. Int J Mol Sci 15: 5446-5457.

45. Ji X, Zhang W, Gui J, Fan X, Zhang W, Li Y, et al. (2014) Role of a genetic variant on the 15q25.1 lung cancer susceptibility locus in smoking-associated nasopharyngeal carcinoma. PLoS One 9: e109036.

46. Nguyen JD, Lamontagne M, Couture C, Conti M, Pare PD, Sin DD, et al. (2014) Susceptibility loci for lung cancer are associated with mRNA levels of nearby genes in the lung. Carcinogenesis 35: 2653-2659.

47. Renieri A, Mencarelli MA, Cetta F, Baldassarri M, Mari F, Furini S, et al. (2014) Oligogenic germline mutations identified in early non-smokers lung adenocarcinoma patients. Lung Cancer 85: 168-174.

48. Dai X, Deng S, Wang T, Qiu G, Li J, Yang B, et al. (2014) Associations between 25 lung cancer risk-related SNPs and polycyclic aromatic hydrocarbon-induced genetic damage in coke oven workers. Cancer Epidemiol Biomarkers Prev 23: 986-996.

49. Hu B, Huang Y, Yu RH, Mao HJ, Guan C, Zhao J. (2014) Quantitative assessment of the influence of common variations (rs8034191 and rs1051730) at 15q25 and lung cancer risk. Tumour Biol 35: 2777-2785.

50. de Mello RA, Ferreira M, Soares-Pires F, Costa S, Cunha J, Oliveira P, et al. (2013) The impact of polymorphic variations in the 5p15, 6p12, 6p21 and 15q25 Loci on the risk and prognosis of portuguese patients with non-small cell lung cancer. PLoS One 8: e72373.

51. Wu H, Wang Y, Wang S, Jiang M, Wang C, Fu W, et al. (2013) Is susceptibility locus for lung cancer in the 15q25 nicotinic acetylcholine receptor gene cluster CHRNA5-A3-B4 associated with risk of gastric cancer? Med Oncol 30: 576.

52. Wu C, Hu Z, Yu D, Huang L, Jin G, Liang J, et al. (2009) Genetic variants on chromosome 15q25 associated with lung cancer risk in Chinese populations. Cancer Res 69: 5065-5072.

53. Walsh KM, Amos CI, Wenzlaff AS, Gorlov IP, Sison JD, Wu X, et al. (2012) Association study of nicotinic acetylcholine receptor genes identifies a novel lung cancer susceptibility locus near CHRNA1 in African-Americans. Oncotarget 3: 1428-1438.

54. Walsh KM, Gorlov IP, Hansen HM, Wu X, Spitz MR, Zhang H, et al. (2013) Fine-mapping of the 5p15.33, 6p22.1-p21.31, and 15q25.1 regions identifies functional and histology-specific lung cancer susceptibility loci in African-Americans. Cancer Epidemiol Biomarkers Prev 22: 251-260.

55. Lan Q, Hsiung CA, Matsuo K, Hong YC, Seow A, Wang Z, et al. (2012) Genome-wide association analysis identifies new lung cancer susceptibility loci in never-smoking women in Asia. Nat Genet 44: 1330-1335.

56. Fehringer G, Liu G, Pintilie M, Sykes J, Cheng D, Liu N, et al. (2012) Association of the 15q25 and 5p15 lung cancer susceptibility regions with gene expression in lung tumor tissue. Cancer Epidemiol Biomarkers Prev 21: 1097-1104.

57. Bae EY, Lee SY, Kang BK, Lee EJ, Choi YY, Kang HG, et al. (2012) Replication of results of genome-wide association studies on lung cancer susceptibility loci in a Korean population. Respirology 17: 699-706.

58. Chikova A, Bernard HU, Shchepotin IB, Grando SA (2012) New associations of the genetic polymorphisms in nicotinic receptor genes with the risk of lung cancer. Life Sci 91: 1103-1108.

59. Jaworowska E, Trubicka J, Lener MR, Masojc B, Zlowocka-Perlowska E, McKay JD, et al. (2011) Smoking related cancers and loci at chromosomes 15q25, 5p15, 6p22.1 and 6p21.33 in the Polish population. PLoS One 6: e25057.

60. Timofeeva MN, McKay JD, Smith GD, Johansson M, Byrnes GB, Chabrier A, et al. (2011) Genetic polymorphisms in 15q25 and 19q13 loci, cotinine levels, and risk of lung cancer in EPIC. Cancer Epidemiol Biomarkers Prev 20: 2250-2261.

61. Young RP, Hopkins RJ, Whittington CF, Hay BA, Epton MJ, Gamble GD. (2011) Individual and cumulative effects of GWAS susceptibility loci in lung cancer: associations after sub-phenotyping for COPD. PLoS One 6: e16476.

62. Thorgeirsson TE, Geller F, Sulem P, Rafnar T, Wiste A, Magnusson KP, et al. (2008) A variant associated with nicotine dependence, lung cancer and peripheral arterial disease. Nature 452: 638-642.

63. Landi MT, Chatterjee N, Yu K, Goldin LR, Goldstein AM, Rotunno M, et al. (2009) A genome-wide association study of lung cancer identifies a region of chromosome 5p15 associated with risk for adenocarcinoma. Am J Hum Genet 85: 679-691.

64. Wang Y, Broderick P, Webb E, Wu X, Vijayakrishnan J, Matakidou A, et al. (2008) Common 5p15.33 and 6p21.33 variants influence lung cancer risk. Nat Genet 40: 1407-1409.

65. Hung RJ, McKay JD, Gaborieau V, Boffetta P, Hashibe M, Zaridze D, et al. (2008) A susceptibility locus for lung cancer maps to nicotinic acetylcholine receptor subunit genes on 15q25. Nature 452: 633-637.

66. Thorgeirsson TE, Gudbjartsson DF, Surakka I, Vink JM, Amin N, Geller F, et al. (2010) Sequence variants at CHRNB3-CHRNA6 and CYP2A6 affect smoking behavior. Nat Genet 42: 448-453.

67. Broderick P, Wang Y, Vijayakrishnan J, Matakidou A, Spitz MR, Eisen T, et al. (2009) Deciphering the impact of common genetic variation on lung cancer risk: a genome-wide association study. Cancer Res 69: 6633-6641.

68. Timofeeva MN, Hung RJ, Rafnar T, Christiani DC, Field JK, Bickeboller H, et al. (2012) Influence of common genetic variation on lung cancer risk: meta-analysis of 14 900 cases and 29 485 controls. Hum Mol Genet 21: 4980-4995.

69. Amos CI, Gorlov IP, Dong Q, Wu X, Zhang H, Lu EY, et al. (2010) Nicotinic acetylcholine receptor region on chromosome 15q25 and lung cancer risk among African Americans: a case-control study. J Natl Cancer Inst 102: 1199-1205.

70. Gabrielsen ME, Romundstad P, Langhammer A, Krokan HE, Skorpen F (2013) Association between a 15q25 gene variant, nicotine-related habits, lung cancer and COPD among 56,307 individuals from the HUNT study in Norway. Eur J Hum Genet 21: 1293-1299.

71. Wang H, Zhao Y, Ma J, Zhang G, Mu Y, Qi G, et al. (2013) The genetic variant rs401681C/T is associated with the risk of non-small cell lung cancer in a Chinese mainland population. Genet Mol Res 12: 67-73.

72. Ito H, McKay JD, Hosono S, Hida T, Yatabe Y, Mitsudomi T, et al. (2012) Association between a genome-wide association study-identified locus and the risk of lung cancer in Japanese population. J Thorac Oncol 7: 790-798.

73. Hsiung CA, Lan Q, Hong YC, Chen CJ, Hosgood HD, Chang IS, et al. (2010) The 5p15.33 locus is associated with risk of lung adenocarcinoma in never-smoking females in Asia. PLoS Genet 6.

74. Truong T, Hung RJ, Amos CI, Wu X, Bickeboller H, Rosenberger A, et al. (2010) Replication of lung cancer susceptibility loci at chromosomes 15q25, 5p15, and 6p21: a pooled analysis from the International Lung Cancer Consortium. J Natl Cancer Inst 102: 959-971.

75. Schwartz AG, Cote ML, Wenzlaff AS, Land S, Amos CI (2009) Racial differences in the association between SNPs on 15q25.1, smoking behavior, and risk of non-small cell lung cancer. J Thorac Oncol 4: 1195-1201
