# Supplementary figures and images for: The SNP rs931794 in 15q25.1 Is Associated with Lung Cancer Risk: A Hospital-Based Case-Control Study and Meta-Analysis
Source: PLoS One. 2015 Jun 16;10(6):e0128201. doi: 10.1371/journal.pone.0128201 (PMC4469418; doi:10.1371/journal.pone.0128201)

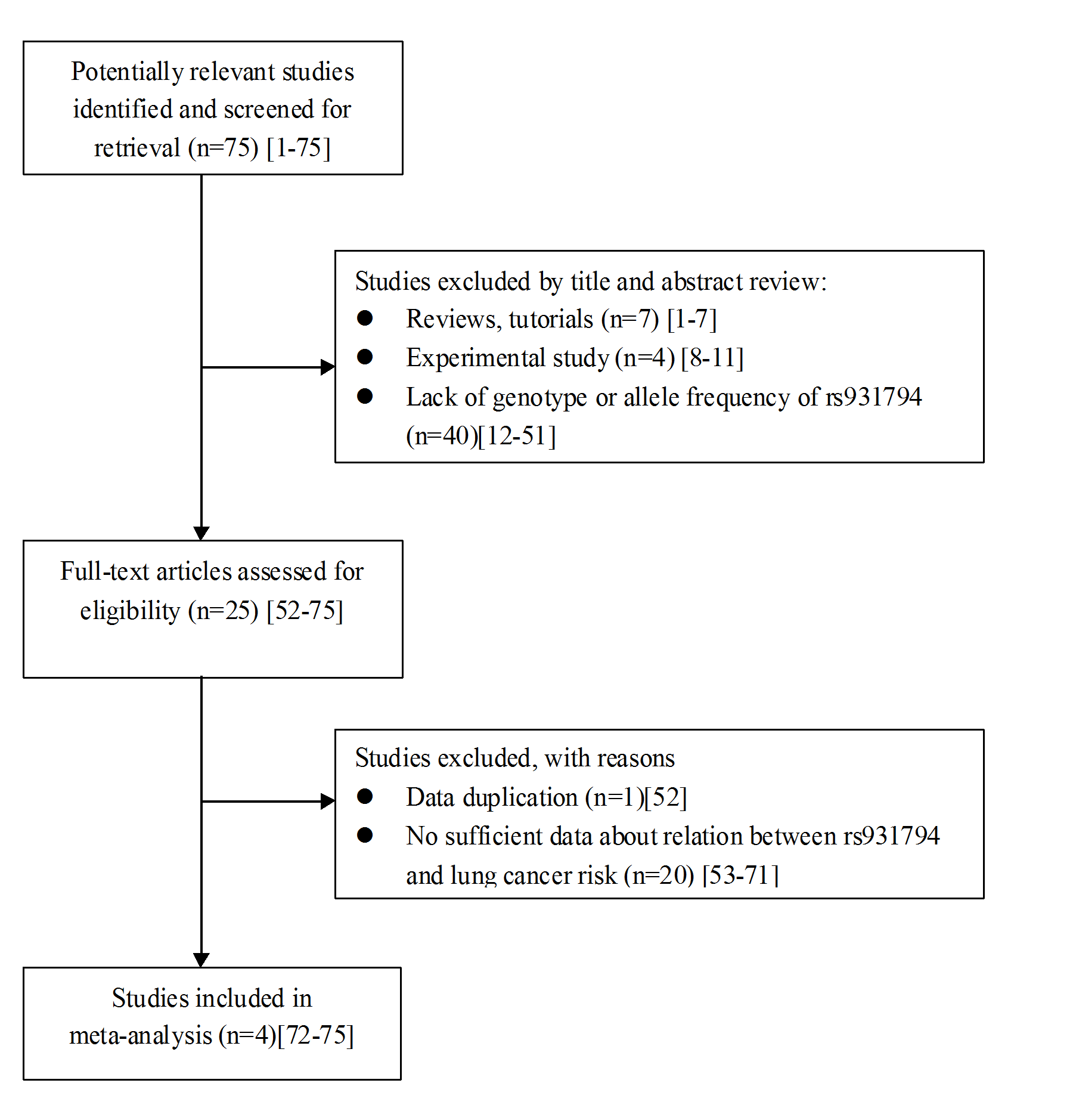

Supplement: S1 Fig — (TIF) [file pone.0128201.s002.tif]

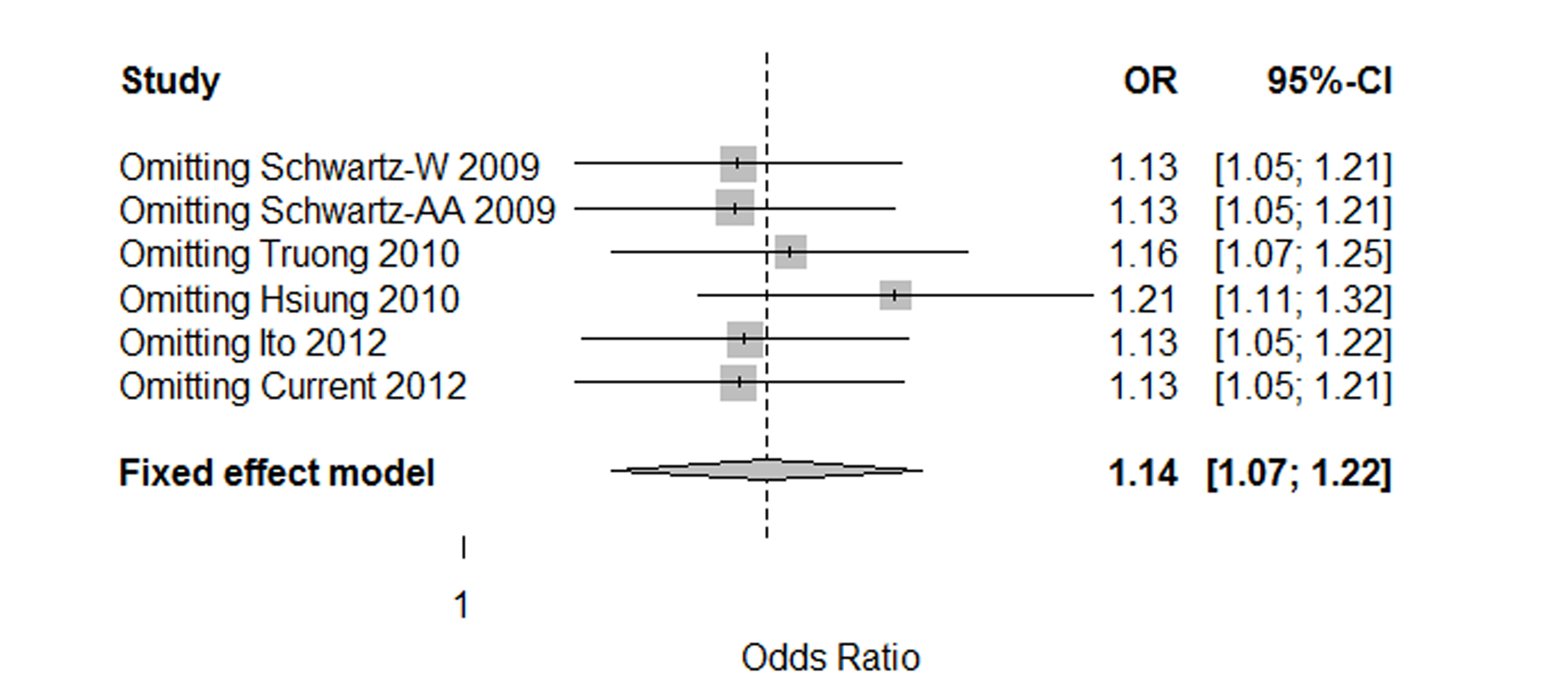

Supplement: S2 Fig — (TIF) [file pone.0128201.s003.tif]
