# Supplementary material for: The SNP rs931794 in 15q25.1 Is Associated with Lung Cancer Risk: A Hospital-Based Case-Control Study and Meta-Analysis
Source: PLoS One. 2015 Jun 16;10(6):e0128201. doi: 10.1371/journal.pone.0128201 (PMC4469418; doi:10.1371/journal.pone.0128201)
Supplement: S2 Table — (DOCX) [file pone.0128201.s005.docx]

Table S2. The association between rs931794 and risk of lung cancer by smoking status, median age, age range and sex.

| Subjects | Genotype | Control | Case | *P^†^* | Additive OR (95%CI) | *p* |
| --- | --- | --- | --- | --- | --- | --- |
| Smoking status |  |  |  |  |  |  |
| Smokers | AA | 298 | 106 | 0.042 | 1.314(1.061-1.627) | 0.012 |
|  | AG | 238 | 113 |  |  |  |
|  | GG | 61 | 37 |  |  |  |
| Non-smokers | AA | 203 | 126 | 0.007 | 1.237(0.998-1.534) | 0.053 |
|  | AG | 211 | 122 |  |  |  |
|  | GG | 42 | 50 |  |  |  |
| Median age |  |  |  |  |  |  |
| Age≤61.0 | AA | 264 | 133 | 0.024 | 1.264(1.025-1.557) | 0.028 |
|  | AG | 218 | 116 |  |  |  |
|  | GG | 48 | 45 |  |  |  |
| Age>61.0 | AA | 239 | 102 | 0.033 | 1.320(1.064-1.639) | 0.012 |
|  | AG | 234 | 123 |  |  |  |
|  | GG | 55 | 43 |  |  |  |
| Age range |  |  |  |  |  |  |
| Age≤50.0 | AA | 73 | 41 | 0.294 | 1.378(0.919-2.067) | 0.121 |
|  | AG | 53 | 40 |  |  |  |
|  | GG | 10 | 11 |  |  |  |
| Age>50.0 | AA | 430 | 194 | 0.002 | 1.283(1.091-1.508) | 0.003 |
|  | AG | 399 | 199 |  |  |  |
|  | GG | 93 | 77 |  |  |  |
| Sex |  |  |  |  |  |  |
| Male | AA | 343 | 162 | 0.001 | 1.294(1.078-1.553) | 0.006 |
|  | AG | 334 | 159 |  |  |  |
|  | GG | 65 | 63 |  |  |  |
| Female | AA | 160 | 73 | 0.120 | 1.271(0.977-1.655) | 0.074 |
|  | AG | 118 | 80 |  |  |  |
|  | GG | 38 | 25 |  |  |  |

†P values were calculated by the Pearson Chi-Square test.

The additive model used in calculating OR.
