# Supplementary material for: The SNP rs931794 in 15q25.1 Is Associated with Lung Cancer Risk: A Hospital-Based Case-Control Study and Meta-Analysis
Source: PLoS One. 2015 Jun 16;10(6):e0128201. doi: 10.1371/journal.pone.0128201 (PMC4469418; doi:10.1371/journal.pone.0128201)
Supplement: S3 Table — (DOCX) [file pone.0128201.s006.docx]

Table S3. Quality assessment of included studies

| Study | Selection | | | | Comparability | | | Exposure | | | Score |
| --- | --- | --- | --- | --- | --- | --- | --- | --- | --- | --- | --- |
|  | Cases | Cases | Community | Controls | | Age-matched | Other | Detection | Same | Similar |  |
|  | independently | representative | controls | have no | | control | factors-matched | of exposure | method for | un-response |  |
|  | validated | of population |  | history of | |  | control | by blinded | detection | rate between |  |
|  |  |  |  | lung | |  |  | interview | between | cases and |  |
|  |  |  |  | cancer | |  |  |  | cases and | controls |  |
|  |  |  |  |  |  |  |  |  | controls |  |  |
| Schwartz | 0 | 1 | 1 | 1 | | 1 | 1 | 0 | 1 | 0 | 6 |
| Truong | 0 | 1 | 0 | 1 | | 1 | 1 | 0 | 1 | 1 | 6 |
| Hsiung | 0 | 1 | 1 | 1 | | 1 | 1 | 0 | 1 | 1 | 7 |
| Ito | 1 | 1 | 0 | 1 | | 1 | 1 | 0 | 1 | 0 | 6 |
| Current Study | 1 | 1 | 0 | 1 | | 1 | 1 | 0 | 1 | 1 | 7 |
