# Supplementary material for: The SNP rs931794 in 15q25.1 Is Associated with Lung Cancer Risk: A Hospital-Based Case-Control Study and Meta-Analysis
Source: PLoS One. 2015 Jun 16;10(6):e0128201. doi: 10.1371/journal.pone.0128201 (PMC4469418; doi:10.1371/journal.pone.0128201)
Supplement: S4 Table — (DOCX) [file pone.0128201.s007.docx]

Table S4. Egger’s test and Nonparametric trim and fill analysis of publication bias in meta-analysis.

| Category | Genetic model | Egger’s test | | | | OR(95%CI)^a^ | | | *P^a^* | | | *P^a^* for heterogeneity | | | | Number of filled studies | | |
| --- | --- | --- | --- | --- | --- | --- | --- | --- | --- | --- | --- | --- | --- | --- | --- | --- | --- | --- |
| Overall | AG/AA | 0.013 | | | | 1.102(1.033-1/175) | | | 0.003 | | | 0.377 | | | | 3 | | |
| (n=6) | GG/AA | 0.065 | | | | -- | | | -- | | | -- | | | | -- | | |
|  | Dominant | <0.001 | | | | 1.100(1.001-1.208) | | | 0.048 | | | 0.032 | | | | 3 | | |
|  | Recessive | 0.100 | | | | -- | | | -- | | | -- | | | | -- | | |
|  | G/A | 0.005 | | | | 1.047(0.946-1.159) | | | 0.377 | | | <0.001 | | | | 3 | | |
|  | Additive | 0.006 | | | | 1.046(0.948-1.154) | | | 0.374 | | | <0.001 | | | | 3 | | |
| *Ethnicity* **/** *Histological type* | | |  |  |  | |  |  | | |  | |  | |  | |  |  |
| Asian (n=4) **/** | AG/AA | 0.125 | | | | --- | | | -- | | | -- | | | | -- | | |
| Lung cancer | GG/AA | 0.042 | | | | 0.967(0.712-1.314) | | | 0.831 | | | <0.001 | | | | 2 | | |
| (n=4) | Dominant | 0.005 | | | | 1.066(0.997-1.140) | | | 0.060 | | | 0.092 | | | | 2 | | |
|  | Recessive | 0.054 | | | | -- | | | -- | | | -- | | | | -- | | |
|  | G/A | 0.031 | | | | 1.015(0.899-1.147) | | | 0.808 | | | <0.001 | | | | 2 | | |
|  | Additive | 0.031 | | | | 1.015(0.901-1.143) | | | 0.809 | | | <0.001 | | | | 2 | | |
| *Genotyping method* | |  | | | |  | | |  |  | |  | |  |  |  |  |  |
| Taqman | AG/AA | 0.032 | | | | 1.109(1.028-1.197) | | | 0.017 | | | 0.284 | | | | 2 | | |
| (n=5) | GG/AA | 0.128 | | | | -- | | | -- | | | -- | | | | -- | | |
|  | Dominant | <0.001 | | | | 1.093(0.972-1.228) | | | 0.136 | | | 0.011 | | | | 3 | | |
|  | Recessive | 0.180 | | | | -- | | | -- | | | -- | | | | -- | | |
|  | G/A | 0.018 | | | | 1.043(0.921-1.182) | | | 0.507 | | | <0.001 | | | | 3 | | |
|  | Additive | 0.020 | | | | 1.042(0.924-1.176) | | | 0.501 | | | <0.001 | | | | 3 | | |

^a^ Data were calculated by nonparametric trim and fill analysis.
